# ORF Organization and Gene Recognition in the Yeast Genome

**DOI:** 10.1002/cfg.292

**Published:** 2003-06

**Authors:** Liaofu Luo, Hong Li, Lirong Zhang

**Affiliations:** Laboratory of Theoretical Biophysics Faculty of Science and Technology Inner Mongolia University Hohhot 010021 China

## Abstract

Some rules on gene recognition and ORF organization in the *Saccharomyces cerevisiae* genome are demonstrated by statistical analyses of sequence data. This study includes: (a) The random frame rule—that the six reading frames W1, W2, W3, C1, C2 and C3 in the double-stranded genome are randomly occupied by ORFs (related phenomena on ORF overlapping are also discussed). (b) The inhomogeneity rule—coding and non-coding ORFs differ in inhomogeneity of base composition in the three codon positions. By use of the inhomogeneity index (IHI), one can make a distinction between coding (IHI > 14) and non-coding (IHI ≤ 14) ORFs at 95% accuracy. We find that ‘spurious’ ORFs (with IHI ≤ 14) are distributed mainly in three classes of ORFs, namely, those with ‘similarity to unknown proteins’, those with ‘no similarity’, or ‘questionable ORFs’. The total number of spurious ORFs (which are unlikely to be regarded as coding ORFs) is estimated to be 470. (c) The evaluation of ORF length distribution shows that below 200 amino acids the occurrence of ATG initiator ORFs is close to random.


**Comparative and Functional Genomics**
*Comp Funct Genom* 2003; **4**: 318–328.
Published online in Wiley InterScience (www.interscience.wiley.com). **DOI:** 10.1002/cfg.292

**Primary Research Paper**

### ORF organization and gene recognition in the yeast genome

Liaofu Luo*, Hong Li and Lirong Zhang
*Laboratory of Theoretical Biophysics, Faculty of Science and Technology, Inner Mongolia University, Hohhot 010021, China*

**Correspondence to: Liaofu Luo, Laboratory of Theoretical Biophysics, Faculty of Science and Technology, Inner Mongolia University, Hohhot, 010021, China. E-mail: lfluo@mail.imu.edu.cn*

Received: 31 October 2002
Revised: 3 March 2003
Accepted: 10 March 2003

### Abstract

**Some rules on gene recognition and ORF organization in the *Saccharomyces cerevisiae* genome are demonstrated by statistical analyses of sequence data. This study includes: (a) The random frame rule — that the six reading frames W1, W2, W3, C1, C2 and C3 in the double-stranded genome are randomly occupied by ORFs (related phenomena on ORF overlapping are also discussed). (b) The inhomogeneity rule — coding and non-coding ORFs differ in inhomogeneity of base composition in the three codon positions. By use of the inhomogeneity index (IHI), one can make a distinction between coding (IHI > 14) and non-coding (IHI ≤ 14) ORFs at 95% accuracy. We find that 'spurious' ORFs (with IHI ≤ 14) are distributed mainly in three classes of ORFs, namely, those with 'similarity to unknown proteins', those with 'no similarity', or 'questionable ORFs'. The total number of spurious ORFs (which are unlikely to be regarded as coding ORFs) is estimated to be 470. (c) The evaluation of ORF length distribution shows that below 200 amino acids the occurrence of ATG initiator ORFs is close to random.** **Copyright © 2003 John Wiley & Sons, Ltd.**

**Keywords:** **yeast genome; open reading frame; random frame; inhomogeneity index; spurious ORFs**

### Introduction

The brewer's yeast (*Saccharomyces cerevisiae*) genome was the first eukaryotic genome sequence to be completed (Mewes *et al.*, 1997; Goffeau *et al.*, 1996). Many theoretical investigations have been carried out to search for the information hidden in the sequence. (Dujon, 1996; Dujon *et al.*, 1994; Termier *et al.*, 1996; Velculescu *et al.*, 1997; Mackiewicz *et al.*, 1999; Blandin *et al.*, 2000; Cliften *et al.*, 2001; Wood *et al.*, 2001). One of the central problems in bioinformatics is gene recognition (International Humun Genome Sequencing Consortium, 2001; Stormo, 2000; Guigo *et al.*, 2000; Burge and Karlin, 1998, 1997; Claverie, 1997; Gelfand *et al.*, 1996; Burset and Guigo, 1996; Krogh *et al.*, 1994). There are three main approaches for gene identification: direct evidence of transcription provided by ESTs or mRNAs; indirect evidence based on sequence similarity to previously identified genes and proteins; and *ab initio* recognition of exons. Theoretical predictions are based mainly on the latter two approaches. For brewer's yeast, where introns are scarce and short, gene prediction is relatively simple; *ab initio* methods alone identify protein-coding genes with relatively high accuracy (International Human Genome Sequencing Consortium, 2001). Various coding measures such as codon usage vectors, base composition vectors and Fourier transform of the sequence have been assessed (Fickett and Tung, 1992; Fickett, 1982; Staden, 1984; Guigo *et al.*, 2000; Stormo, 2000). However, the essence of the coding potential of a DNA sequence is still unclear at present. Here we assess how this problem is related to ORF organization. Brewer's yeast contains thousands of ORFs that have been well identified and investigated. In this work the following points were addressed with respect to *Saccharomyces cerevisiae*:

Copyright © 2003 John Wiley & Sons, Ltd.

1. Biologists currently have a model that there is no distributional bias in the location of ORFs with respect to frame position (Dujon, 1996). The limitation of the observation of 'no bias' was addressed. We assessed whether the coding frame stochastically occupied by an ORF influences the frame occupied by adjacent ORFs and applied direct statistics to the distribution of ORFs in the six frames of the yeast genome.
2. Although six ORF frames occur stochastically in a DNA sequence, we have demonstrated that, among other factors, the coding potential of a DNA segment should satisfy some necessary condition, viz. the asymmetry of base composition in three codon positions. Based on statistical theory, we have defined an inhomogeneity index (IHI) for a segment of DNA sequence. By use of this IHI as a protein-coding measure, protein-coding sequences can be differentiated from non-protein-coding sequences with high accuracy. Moreover, in contrast to other algorithms, no special training process is necessary in this approach for the discrimination of coding/non-coding segments. We have shown that, for brewer's yeast, the accuracy of gene identification by use of this method has attained 95%. The universality of the IHI function will also be indicated briefly. By application of the same IHI rule to other microorganism genomes, we can identify protein-coding genes at an accuracy >90%.
3. To study the distribution of ORF length we defined an ideal ORF as any ORF of one codon or more with an initiator ATG. More plainly, the ideal ORF is defined by a DNA segment from the first ATG after the nearest terminator to a downstream stop codon in the same frame. The number of ideal ORFs in each length interval was calculated and compared with the real ORF length distribution. We found that in the range of size <150 amino acids, the number of ideal ORFs in the *S. cerevisiae* genome is explicitly higher than the number of real ORFs in the databases. In addition, the distribution of ideal ORFs with length <200 amino acids is very close to that in stochastic sequences. This shows that below 200 amino acids, the occurrence of ATG initiator ORFs is close to random. The chief aim of this study is to indicate that the above three rules — the random frame rule, the inhomogeneity rule and the random occurrence of short ORFs — hold well and may be used for the interpretation of ORF organization and gene recognition in the *S. cerevisiae* genome.

### Methods

### Database

The sequence of the *Saccharomyces cerevisiae* genome was downloaded from GenBank on 2 April 2002 (**ftp://ncbi.nlm.nih.gov/genbank**). The ORFs can be divided into six classes: known proteins; strong similarity to known proteins; similarity or weak similarity to known proteins; similarity to unknown proteins; no similarity; and questionable ORFs. The above classifications were downloaded from MIPS (**http://mips.gsf.de/proj/yeast**) issued on 30 October 2001 and renewed in April 2002. Following the name of each ORF given in MIPS, we searched for the corresponding sequence data in GenBank and performed the calculation. The total number of ORFs in the MIPS/GenBank matching database is 5950 for intron-less, 29 for mitochondrial and 237 for intron-containing ORFs.

### Frame orientation statistics

The frame used for defining an ORF is denoted by W1, W2, W3, C1, C2 or C3, where W and C mean the positive and negative strand, respectively, and 1, 2 and 3 indicate the three possible reading frames occupied by the first nucleotide of the ORF relative to the strand initiation site of the chromosome. Setting the first nucleotide of an ORF as being on the $(3N + j)$th site (where $N =$ an integer, $j = 1, 2, 3$; $j = 3$ equivalent to $j = 0$) relative to the strand initiation, one defines the ORF as in the frame W$j$ (for ORFs on the W strand) or in the frame C$(j + 1)$ (for ORFs on the C strand). For example, the ORF on the W strand at sites 109 433–109 960 is in the W2 frame; the ORF on the C strand at bases 219 190–220 191 is in the C1 frame. Evidently, frames 1, 2 and 3 shifted by one base are coincident with frames 2, 3 and 1, respectively. So, a chromosome can be described by a sequence of ORFs written by six letters — W1, W2, W3, C1, C2 and C3, successively. To describe the deviation from homogeneity

Copyright © 2003 John Wiley & Sons, Ltd. 

of frames occupied by ORFs in a chromosome, one may introduce:

$$D_1 = (H_{\max} - H)/H_{\max}, \quad H = -\Sigma p_j \log_2 p_j \tag{1}$$

where $p_j$ is the probability of $j$th frame ($j = 1, \ldots, 6$) occurring in the chromosome, and $H_{\max} = \log_2 6$. To describe the deviation from independence of neighbouring frames in a chromosome one may introduce:

$$D_2 = (2H - H_{\text{joint}})/H_{\max}, \quad H_{\text{joint}} = -\Sigma p_{ji} \log_2 p_{ji} \tag{2}$$

where $p_{ji}$ is the joint probability of a pair of frames — the $j$th and the $i$th frame — occurring in the successive positions of a chromosome. When the frames are randomly distributed in a chromosome, one has $D_1 = D_2 = 0$ as chromosome length approaches infinity.

Gene overlapping is an interesting phenomenon in the genomes of lower organisms (Fukuda *et al.*, 1999). Overlapping genes are defined as a pair of adjacent genes whose coding regions are partly overlapping. There are three kinds of overlapping genes, overlapping in the W strand, overlapping in the C strand (both unidirectional overlaps) and, overlapping in the W and C strands (bidirectional overlapping). The numbers of the three kinds of overlapping genes were obtained from direct enumeration in each chromosome. Note that if a shorter ORF overlaps fully with a part of another longer ORF in the same frame, then the shorter one was not enumerated in this study (Dujon *et al.*, 1994; Mackiewicz *et al.*, 1999). However, shorter ORFs contained in alternative reading frames of the same strand or in any of the three reading frames of the opposite strand should be enumerated as well.

### Definition of inhomogeneity index

To identify an ORF as being coding or non-coding we used a quantity based on Pearson's statistics (Feller, 1971), called the inhomogeneity index (IHI). The IHI of an ORF, or a segment of sequence, is defined as follows (Lee and Luo, 1997a): let $N_j$ be the number of base $j$ ($j$ = A,C,G,T) in a segment (length $N$) of DNA sequence. We divide the segment into $N/m$ multiplets with $m$ bases in each multiplet. Let $N_{ja}$ be the number of base $j$ in the $a$th ($a = 1, 2, \ldots, m$) position of the multiplets and:

$$\text{IHI} = \sum_a^m \sum_j \frac{\left(N_{ja} - \frac{N_j N_a}{N}\right)^2}{\frac{N_j N_a}{N}}$$

$$N_a = \sum_j N_{ja} = N/m \tag{3}$$

In the present case, the multiplet is the codon and $m = 3$. According to Pearson's theorem, if each base is homogeneously distributed over the three codon positions, then IHI obeys the $\chi^2$ distribution with six degrees of freedom as $N$ is large enough. For a homogeneous sequence segment the expectation value of IHI is 6, with standard deviation $\sqrt{12} = 3.46$. The larger the inhomogeneity is, the greater the IHI will be.

We have previously found that there is a substantial difference in IHI between exons and introns and other non-coding regions (Lee and Luo, 1997a). In this work, the parameter IHI will be used to differentiate between coding ORFs and intergenic sequences. By use of first class ORFs (i.e. known proteins) as positive samples, and intergenic sequences as negative samples, we are able to differentiate between the two sample sets by calculation of IHI values. After removing intron-containing and mitochondrial ORFs, we chose 3081 ORFs in the first class as positive samples. We obtained 3552 intergenic sequences with lengths longer than 300 bp. They comprised the negative samples. For each sample the IHI value was calculated.

### ORF length distribution

The distribution of lengths of intron-less ORFs in the database was compared with that of ideal ORFs in the yeast genome, and in stochastic sequences. The ideal ORFs were found by searching for stop codons, then finding the first ATG downstream of them and obtaining a DNA segment from this ATG to the next terminator in the same frame. So, the ideal ORF initiates from 'the first ATG' and always has a length of multiples of three bases. The stochastic sequence was generated randomly from the four bases A, C, G and T, with length 12.2 million bases, under the component

Copyright © 2003 John Wiley & Sons, Ltd. 

constraints of the yeast genome (i.e. under the same ratio of nucleotide contents, A : C : G : T, as in the *S. cerevisiae* genome).

### Results and discussion

### The randomness and independence of frames

The statistics on the distribution of ORFs in the six frames — W1, W2, W3, C1, C2 and C3 — are given in Table 1. The frequencies of frame-changes between successive ORFs in each chromosome are listed. If there is no distributional bias with respect to frame choice in the W strand or C strand, then the number of frame-changing events (i.e. the probability that the frames taken by two consecutive ORFs are different from each other) should be about two-thirds of the total number of ORFs in a single chain. The statistical results on the percentage of frame-changes in the W strand and C strand are given in the second and third columns of Table 1, respectively. They are distributed between 0.57 and 0.76, and are near to two-thirds, as required by the no-bias assumption. Likewise, if there is no bias with respect to strand selection, then the number of frame-changing events between the plus and minus strand for successive ORFs should be about one-half of the total number of ORFs, as for any given frame the next frame can be on one of the two possible strands, W or C. The statistical results on the percentage of W/C strand changing are listed in last column of Table 1 and they are distributed between 0.46 and 0.56, and are near to one-half, as required by the no-bias assumption.

The randomness of ORF orientation in the brewer's yeast genome was first indicated by Dujon (1996). To check the randomness and independence of the six kinds of ORFs, one may use a six-symbol (W1, W2, W3, C1, C2 and C3) sequence representing the ORF arrangement in a chromosome and calculate its informational redundancies $D1$ (deviation from randomness) and $D2$ (deviation from independence of adjacent ORFs). The results are listed in Table 2. The first-order informational redundancies $D1$ are generally <0.01. The second-order informational redundancies $D2$ are generally <0.1. This indicates that the six frames are occupied randomly by ORFs and the frames of adjacent ORFs are independent. In fact, for a random sequence of six symbols, $D1$ obeys the $\chi^2$ distribution with five degrees of freedom, and $D2$ obeys the $\chi^2$ distribution with 25 degrees of freedom, as sequence length (ORF number in a chromosome) $L \rightarrow \propto$ (Luo and Li, 1991). So, the fluctuation bounds of $D1$ and $D2$ (at confidence level 99%) for any six-symbol sequence can be

**Table 1.** Statistics on the frequency of frame changes between successive ORFs in the *S. cerevisiae* genome

| Chromosome | W strand | C strand | Double strands |
|---|---|---|---|
| 1 | 36/55 (0.655) | 33/50 (0.660) | 50/105 (0.476) |
| 2 | 136/203 (0.670) | 148/232 (0.638) | 203/435 (0.467) |
| 3 | 52/75 (0.693) | 60/98 (0.612) | 98/173 (0.566) |
| 4 | 266/403 (0.660) | 282/425 (0.664) | 425/828 (0.513) |
| 5 | 104/146 (0.712) | 89/143 (0.622) | 147/289 (0.509) |
| 6 | 39/68 (0.574) | 38/66 (0.576) | 66/134 (0.493) |
| 7 | 190/299 (0.635) | 176/274 (0.642) | 274/573 (0.478) |
| 8 | 95/153 (0.621) | 97/131 (0.740) | 154/284 (0.542) |
| 9 | 65/99 (0.657) | 86/116 (0.759) | 99/215 (0.460) |
| 10 | 143/204 (0.701) | 132/187 (0.706) | 204/391 (0.522) |
| 11 | 110/174 (0.632) | 109/164 (0.665) | 164/338 (0.485) |
| 12 | 171/261 (0.655) | 192/287 (0.669) | 262/548 (0.478) |
| 13 | 177/248 (0.714) | 155/245 (0.633) | 248/493 (0.503) |
| 14 | 153/222 (0.689) | 143/201 (0.711) | 222/423 (0.525) |
| 15 | 207/298 (0.695) | 173/278 (0.622) | 299/576 (0.519) |
| 16 | 164/258 (0.636) | 164/243 (0.675) | 259/501 (0.517) |

The ratios a/b give the proportions of frame changes, a = the number of frame changes between successive ORFs; b = the total number of ORFs in given chromosome. The values of each ratio are given in brackets.

**Table 2.** Informational parameters $D1$ and $D2$ for ORF sequence written by six-frame symbols

| Chromosome | $D1$ | $D2$ | Chromosome | $D1$ | $D2$ |
|---|---|---|---|---|---|
| 1 | 0.0149 | 0.1162 | 9 | 0.0056 | 0.0451 |
| 2 | 0.0022 | 0.0189 | 10 | 0.0050 | 0.0258 |
| 3 | 0.0122 | 0.0600 | 11 | 0.0020 | 0.0321 |
| 4 | 0.0016 | 0.0138 | 12 | 0.0038 | 0.0175 |
| 5 | 0.0036 | 0.0265 | 13 | 0.0010 | 0.0222 |
| 6 | 0.0104 | 0.0594 | 14 | 0.0019 | 0.0111 |
| 7 | 0.0042 | 0.0127 | 15 | 0.0011 | 0.0113 |
| 8 | 0.0056 | 0.0350 | 16 | 0.0036 | 0.0227 |

$D1$ = the deviation from homogeneity of frames in a chromosome; $D2$ = the deviation from independence of adjacent frames in a chromosome, see text. All values of $D1$ and $D2$ are lower than the fluctuation bound (see equations 4 and 5 in text). The fluctuation bound depends on $L$ (the number of ORFs in a chromosome), which can be found in the last column (the denominator of the fraction) of Table 1.

Copyright © 2003 John Wiley & Sons, Ltd.

defined as:

$$D1(\text{fluctuation}) = 15.1/(L \ln 6) = 8.43/L \quad (4)$$

$$D2(\text{fluctuation}) = 44.3/(L \ln 6) = 24.7/L \quad (5)$$

(where $L$ means sequence length or ORF number in a chromosome in the present case) by use of the $\chi^2$ distribution. This means, for a six-symbol sequence describing the frames of a chromosome, that if the calculated $D1$ is lower than (4) then it does not conflict with the randomness assumption, and if the calculated $D2$ is lower than (5) then it does not conflict with the independence assumption. Considering $L$, and taking a value of several hundreds ($L$ can be found in the last column of Table 1, i.e. the denominator of fractions in that column) the values of $D1$ and $D2$ given in Table 2 are low enough that they are consistent with the randomness and independence of frames. Further, if we study the first class ORFs only (known proteins), similar calculations give values of $D1 = 0.002–0.015$ and $D2 = 0.04–0.3$, respectively. Since the first-class ORF number is about one-half of the total ORF number of six classes and the first-class ORF-pair number is about one-third to one-quarter of the total ORF-pair number of six classes in a chromosome, the above results on $D1$ and $D2$ are also consistent with the randomness and independence of frames. Note that the number of frame-changes we obtained neglected the effect of intron insertion, as intron length is not always a multiple of 3. However, introns in *S. cerevisiae* are comparatively rare (237 intron-containing ORFs in 6000 genes) and their effect on the number of frame-changes is negligible.

Using similar calculations, we obtain $D1 = 0.00032$ and $D2 = 0.057$ for *E. coli* and $D1 = 0.0011$ and $D2 = 0.068$ for *B. subtilis*. Taking $L = 4289$ for *E. coli* and 4100 for *B. subtilis*, we can demonstrate the randomness of frames in these two genomes. However, the independence of the orientation of adjacent frames has been broken, since $D2$ exceeds the fluctuation bound (equation 5), possibly due to the existence of operons.

Our statistics prove the randomness and independence of frames quantitatively by direct calculations of $D1$ and $D2$ and by the comparison of these quantities with fluctuation bounds. As for the origin of randomness and independence a tentative explanation is: that primary ORF formation is basically a stochastic event in the early stages of genome evolution. It happens at many sites independently. Since there is no known biological requirement for any bias in ORF organization in *S. cerevisiae* the randomness and independence of frames is then retained.

### Statistical analysis of the frequency of overlaps

Unidirectional overlapping of ORFs in the W strand and the C strand and bidirectional overlapping of ORFs between the two strands have been enumerated in each chromosome. The results for the number of overlapping gene pairs are summarized in Table 3. It shows that the overlapping ORFs (490 pairs, or 980 ORFs, in total) constitute about one-sixth of the total numbers of ORFs. The numbers of overlapping pairs in the W and C strands are given in the second and third columns, respectively, of Table 3. They include overlapping pairs in different frames and in the same frame. For the latter, we find that they occur generally in the case of the longer one being intron-containing. Two typical

**Table 3.** Statistics on overlapping gene pairs

| Chromosome | W | C | Total (uni.) | W/C | Total |
|---|---|---|---|---|---|
| 1 | 1 (0) | 0 (0) | 1 (0) | 7 | 8 |
| 2 | 6 (3) | 5 (1) | 11 (4) | 28 | 39 |
| 3 | 4 (1) | 2 (0) | 6 (1) | 11 | 17 |
| 4 | 7 (4) | 6 (4) | 13 (8) | 62 | 75 |
| 5 | 2 (0) | 3 (3) | 5 (3) | 8 | 13 |
| 6 | 2 (1) | 2 (0) | 4 (1) | 3 | 7 |
| 7 | 7 (4) | 4 (2) | 11 (6) | 38 | 49 |
| 8 | 1 (1) | 1 (1) | 2 (2) | 2 | 4 |
| 9 | 2 (1) | 0 (0) | 2 (1) | 2 | 4 |
| 10 | 9 (3) | 3 (0) | 12 (3) | 29 | 41 |
| 11 | 1 (0) | 3 (0) | 4 (0) | 19 | 23 |
| 12 | 4 (2) | 10 (1) | 14 (3) | 37 | 51 |
| 13 | 6 (2) | 4 (2) | 10 (4) | 27 | 37 |
| 14 | 1 (0) | 6 (1) | 7 (1) | 27 | 34 |
| 15 | 11 (3) | 2 (1) | 13 (4) | 32 | 45 |
| 16 | 7 (2) | 5 (2) | 12 (4) | 31 | 43 |
| Total | 71 (27) | 56 (18) | 127 (45) | 363 | 490 |

The second column (W) and the third column (C) give the number of overlapping gene pairs in the W chain and C chain respectively. The fourth column Total (uni.) is the sum of the W and C columns, which gives the number of uni-directional overlapping ORF pairs. The numbers in brackets refer to the overlapping pairs in the same frame. The fifth column (W/C) gives the number of di-directional overlapping ORF pairs. The last column (Total) is the sum of the number of uni-directional overlapping ORF pairs and di-directional overlapping ORF pairs.

Copyright © 2003 John Wiley & Sons, Ltd. 

examples are: YCL019W (85 101–90 414) and YCL020W (85 101–86 417), an overlapping pair in frame W3; and YMR045C (357 358–362 626) and YMR046C (361 304–362 626) an overlapping pair in frame C2. In YCL019W, there are two exons (85 101–86 390 and 86 392–90 414) annotated in the database. In YMR045C there are two exons (357 358–361 320 and 361 322–362 626) annotated in database. The number of these overlaps (in the same frame) is given in brackets in columns two to four of Table 3. They constitute 9.2% of the total number of overlapping pairs. We do not know if there are possible errors in sequencing and annotation for some of these overlaps. Besides the overlap in-frame, other overlapping pairs are related to frame-changing, which include unidirectional overlaps and bidirectional overlaps. The numbers of bidirectional overlaps are shown in the fifth column of Table 3. They are the most important overlap mode, constituting 74% of the total number of overlapping pairs.

### Assessment of coding potential

Fickett *et al.* (1992) proposed four classes of content measures. The first class is based on codon usage (and counts of in-phase words); the second method is related to the encoded amino acid sequence; the third is related to the base compositional bias between codon positions; and the fourth is based on imperfect periodicity in base occurrences, etc. In fact, these measures are related to one another, some methods are slight variants or special cases of others, e.g. the periodicity of coding DNA sequence means a strong resonance at the one-third position of the correlation spectrum (Lee and Luo, 1997b; Lobzin and Chechetkin, 2000). Detailed calculations indicated that the resonance occurs due to the four bases being non-uniformly distributed at the three codon positions (Lee and Luo, 1997b). So, the base compositional bias at the three codon positions is a key feature in the detection of the coding potential of a DNA segment. The information used for gene recognition is usually classified into three types: signals, content measures and similarity measures (Stormo, 2000). The ATG initiator and the splice site in intron–exon boundaries are examples of signal. The IHI parameter (inhomogeneity index) defined above (equation 3) is an example of content measures. Methods based on content measures combined with methods based on signals provide an efficient approach to gene recognition, with high accuracy (Burge and Karlin, 1998). The IHI is important since, based on the Pearson theorem, it can differentiate between random and non-random occurrence of the four bases at the three codon positions and can therefore describe the base compositional bias using an elaborate argument. It can serve as a marker to differentiate between coding and non-coding ORFs. In combination with other methods, this method may provide an additional tool for assessing the coding potential for an ORF, on a gene-by-gene basis. We used the index to study the yeast genome, and the results are summarized in Table 4. The distribution of IHI ranges is very different for known proteins (the first class ORFs) compared to intergenic sequences. The boundary marker can be defined as IHI = 14. More than 95% of positive samples had an IHI > 14. Only 152 positive samples (4.9% of 3081) had an IHI $\leq$ 14. More than 95% of negative samples had an IHI $\leq$ 14, and only 171 negative samples (4.8% of 3552) had IHI > 14. So, gene identification using the IHI rule (using IHI = 14 as a boundary marker) has a sensitivity, $S_n$, of 0.951

**Table 4.** The distribution of IHI in first class ORFs and intergenic sequences

**First class ORFs**

| IHI range | ORF no. | IHI range | ORF no. | IHI range | ORF no. |
|---|---|---|---|---|---|
| (0–7) | 30 | (77–84) | 125 | (147–154) | 33 |
| (7–14) | 122 | (84–91) | 124 | (154–161) | 32 |
| (14–21) | 199 | (91–98) | 103 | (161–168) | 19 |
| (21–28) | 243 | (98–05) | 85 | (168–175) | 22 |
| (28–35) | 241 | (105–112) | 89 | (175–182) | 18 |
| (35–42) | 225 | (112–119) | 74 | (182–189) | 22 |
| (42–49) | 253 | (119–126) | 62 | (189–196) | 16 |
| (49–56) | 216 | (126–133) | 47 | (196–203) | 19 |
| (56–63) | 175 | (133–140) | 46 | (203–210) | 12 |
| (63–70) | 166 | (140–147) | 39 | >210 | 111 |
| (70–77) | 113 | | | | |

**Intergenic sequences**

| IHI range | Seq. no. | IHI range | Seq. no. | IHI range | Seq. no. |
|---|---|---|---|---|---|
| (0–1) | 57 | (5–6) | 432 | (10–11) | 120 |
| (1–2) | 241 | (6–7) | 350 | (11–12) | 95 |
| (2–3) | 366 | (7–8) | 278 | (12–13) | 78 |
| (3–4) | 463 | (8–9) | 202 | (13–14) | 43 |
| (4–5) | 482 | (9–10) | 169 | >14 | 171 |

$(a - b) = a < \text{IHI} \leq b$.

Copyright © 2003 John Wiley & Sons, Ltd.

and a specificity, $S_p$, of 0.945. The accuracy (the average of $S_n$ and $S_p$) is 94.8%. The IHI for a homogeneous sequence is distributed in the range with expectation value 6 and standard deviation 3.46, according to $\chi^2$ distribution. The negative sample having IHI $\leq 14$ means that the intergenic sequence behaves as homogeneous within two standard deviations. This demonstrates that symmetry of base composition among the three codon positions is a feature of the majority of intergenic sequence and that the coding potential of a DNA segment is closely related to the inhomogeneity of its base distribution.

### Detection of spurious ORFs

The detection of spurious ORFs in the yeast genome has attracted the attention of many authors (Termier and Kalogeropoulos, 1996; Velculescu *et al.*, 1997; Cliften *et al.*, 2001; Wood *et al.*, 2001). The IHI parameter is a rapid and simple tool to distinguish coding and non-coding ORFs with high accuracy. We have calculated the IHI for each intron-less ORF in the MIPS/GenBank yeast database; the results are shown in Table 5. (The IHI values for intron-containing ORFs are scattered; the more the intron number and the larger the intron length, the lower the IHI, so we have ignored them in our statistical analysis.) The total number of ORFs in each class and the number of IHI $> 14$ and IHI $\leq 14$ ORFs are listed in the second, third and fourth line of the table, respectively. Of the 5950 ORFs there are the 5187 with IHI $> 14$ and 763 with IHI $\leq 14$. As expected, the percentage of IHI $\leq 14$ ORFs in the six classes (line 5 of Table 5) increases from the first class to the sixth class. Considering the 95% confidence level of IHI as a tool to distinguish coding and non-coding ORFs, we can estimate the number of 'spurious' ORFs (unlikely to be regarded as coding) from the percentage of IHI $\leq 14$ ORFs in each class. The results are shown in the last line of Table 5, e.g. for the sixth class one has $[385 \times (53.5 - 4.93)\% = 187]$ spurious ORFs. The spurious ORF number in the first class is assumed to be zero. So, in the above calculation the IHI $\leq 14\%$ has 4.93% subtracted, which is attributed to false identification due to the 95% confidence level of IHI. As seen from Table 5, the spurious ORFs can be neglected in the second class. They are distributed mainly in last three classes, viz. similarity to unknown proteins, no similarity or questionable ORFs. The total number of spurious ORFs is estimated to be 470, which is close to the recent estimation of Wood *et al.* (2001).

The distribution of IHI $\leq 14$ ORFs in the 16 chromosomes of yeast is given in Table 6. The percentage of ORFs with IHI $\leq 14$ is near 13%. Considering that the spurious ORFs existed mainly in the fourth, fifth and sixth classes we calculated the ratio ($r$) of the number of ORFs with IHI $\leq 14$ to the number of ORFs in classes 4, 5 and 6. It is near 0.4 for most chromosomes, but for chromosomes 2 and 3 the ratio is $>0.5$ and for chromosome 14 it is $<0.3$. The spurious ORF density seems different for different chromosomes. A full list of the names of all yeast ORFs with IHI $\leq 14$ is provided as supplementary material (see: **http://www.interscience.wiley.com/jpages/1531-6912/sites.html**).

The IHI defined by us can be applied to other genomes. Using the same rule for *E. coli* we found that more than 92.3% of positive samples had IHI $> 14$ and more than 93.6% of negative samples had IHI $\leq 14$. For *B. subtilis*, we have shown that $>90.6\%$ of positive samples have IHI $>$ 15 and $>91.5\%$ of negative samples have IHI $\leq$ 15. So, the IHI rule appears to be valid at least for some prokaryotic organisms, in addition to brewer's yeast. Historically, other measures based on compositional bias have been proposed. For example, after defining the frequency of base $j$ in position $a$ ($a = 1, 2, 3$) as $f(j, a)$, one may introduce three measures, $M1$, $M2$ and $M3$ (Fickett and Tung, 1992; Fickett, 1982; Staden, 1984), as

**Table 5.** The number of ORFs with IHI $> 14$ and IHI $\leq 14$ in six classes

| Class | 1 | 2 | 3 | 4 | 5 | 6 | Total |
|---|---|---|---|---|---|---|---|
| ORFs | 3081 | 219 | 818 | 954 | 493 | 385 | 5950 |
| IHI > 14 | 2929 | 208 | 767 | 792 | 312 | 179 | 5187 |
| IHI ≤ 14 | 152 | 11 | 51 | 162 | 181 | 206 | 763 |
| IHI ≤ 14(%) | 4.93 | 5.02 | 6.23 | 17.0 | 36.7 | 53.5 | 12.8 |
| Spurious | 0 | 0.2 | 10.6 | 115 | 157 | 187 | 470 |

The second row, ORFs, indicates the number of ORFs in each class (except intron-containing and mitochondrial ORFs; see text). ORFs are taken from the MIPS database and GenBank. The ORF numbers with IHI $> 14$ and IHI $\leq 14$ are given in third and fourth rows respectively. The percentages of ORFs with IHI $\leq 14$ are listed in fifth line. The estimated spurious ORF numbers for each class are listed in the last line. The spurious ORF number for a given class is estimated as follows: (IHI $\leq 14(\%) - 4.93) \div 100 \times$ (ORF number).

Copyright © 2003 John Wiley & Sons, Ltd. 

**Table 6.** The distribution of ORFs with IHI ≤ 14 in the 16 *S. cerevisiae* chromosomes

| Chrom | 1 | 2 | 3 | 4 | 5 | 6 | 7 | 8 | 9 | 10 | 11 | 12 | 13 | 14 | 15 | 16 | Total |
|---|---|---|---|---|---|---|---|---|---|---|---|---|---|---|---|---|---|
| ORFs | 96 | 407 | 166 | 780 | 269 | 125 | 546 | 264 | 201 | 372 | 327 | 516 | 450 | 404 | 553 | 474 | 5950 |
| IHI ≤ 14 | 13 | 63 | 29 | 98 | 29 | 16 | 75 | 32 | 20 | 48 | 37 | 83 | 55 | 40 | 69 | 56 | 763 |
| (%) | 13.5 | 15.5 | 17.5 | 12.6 | 10.8 | 12.8 | 13.7 | 12.1 | 10.0 | 12.9 | 11.3 | 16.1 | 12.2 | 9.9 | 12.5 | 11.8 | 12.8 |
| r | 0.41 | 0.55 | 0.71 | 0.42 | 0.50 | 0.43 | 0.41 | 0.44 | 0.36 | 0.40 | 0.37 | 0.46 | 0.40 | 0.29 | 0.38 | 0.38 | 0.42 |

The third and fourth rows give the number and percentage of ORFs with IHI ≤ 14 in each chromosome. The fifth line (r) gives the ratio of the number of ORFs with IHI ≤ 14 to the number of ORFs in classes 4, 5 and 6.

follows.

$$M1 = \sum_j \frac{\max(f(j,1), f(j,2), f(j,3))}{1 + \min(f(j,1), f(j,2), f(j,3))}$$

$$M2 = \sum_{j,a} \left| f(j,a) - \frac{f(j,1) + f(j,2) + f(j,3)}{3} \right|$$

$$M3 = \sum_{j,a} \left( f(j,a) - \frac{f(j,1) + f(j,2) + f(j,3)}{3} \right)^2 \quad (6)$$

Consider chromosome 1 of *S. cerevisiae* as an example; it contains 245 positive samples and 236 negative samples have been defined. Through calculation of $M1$, $M2$ and $M3$ for each sample, we obtain 90% of positive samples having $M1 > 0.945$ and 90% of negative samples having $M1 \leq 0.945$, 88.6% of positive samples having $M2 > 0.355$ and 88.6% of negative samples having $M2 \leq 0.355$, and 88.2% of positive samples having $M3 > 0.015$ and 87.3% of negative samples having $M3 \leq 0.015$. The results show that we can use $M1$, $M2$ and $M3$ as protein-coding measures, but their accuracies are 90%, 88.8% and 88%, respectively, lower than IHI by about 5–7 points. So, IHI is a better parameter to measure the coding potential of a sequence. As compared with the 'YZ score' method proposed by Zhang and Wang (2000), both methods use the information on the frequency of the four bases at the three codon positions. However, the IHI, defined by a non-linear function of $N_{ja}$, reflects the essence of the difference of coding and non-coding sequences in a simple formula and no learning is necessary in its application. It seems simpler and more easily manipulated than the YZ score (in the YZ score, the linear discriminant equation for 10 functions of $N_{ja}$ was used, and the Fisher's coefficients were determined empirically). Despite the success of IHI in the classification of coding and non-coding sequences for yeast and lower organisms, its application to higher organisms to determine coding or non-coding sequence is more difficult, due to the presence of many large introns, small exons and large intergenic regions. However, in combination with other methods, the generalization of IHI may provide an additional tool for gene recognition, which will be useful in genome informatics.

Copyright © 2003 John Wiley & Sons, Ltd.

### Evaluation of ORF length distribution

To study the distribution of ORF lengths in the *S. cerevisiae* genome, we have compared it with that of ideal ORFs. Ideal ORFs are deduced theoretically, from a single principle, assuming only that they initiate from the first in-frame ATG codon in the DNA sequence after the nearest upstream stop codon, and no other constraints are supposed. They are different from real ORFs (those annotated in the databases) in two respects: first, they may have very short length, since no size limitation is presupposed; and second, they have length of multiples of 3, since any ideal ORF should terminate at an in-frame stop codon. Nearly all intron-less ORFs (99.7%) in the databases have been found in the set of ideal ORFs deduced above. However, if an intron with a length that is not a multiple of 3 exists, then the corresponding intron-containing gene cannot be recognized by this approach. However, due to the scarcity of introns in the yeast genome the statistics on the distribution of lengths of ideal large-size ORFs will be near to that of real ORFs. The total number of ideal ORFs in the 16 chromosomes of yeast in all six frames is about $2.8 \times 10^5$. The numbers of ideal ORFs in each length interval are shown in Figure 1A. The numbers of real intron-less ORFs in the databases for each length interval are also plotted. From this figure we find that the difference between the two distributions is very small and the two curves are nearly coincident as length becomes >200–230 amino acids. However, in the range of length <150 amino acids the numbers of ideal ORFs are explicitly higher than the numbers of intron-less ORFs in the databases.

To understand the meaning of the statistics on ideal ORF lengths (i.e. the data in Figure 1A), we generated stochastic sequences under the component constraints of the yeast genome. We then obtained all ideal ORFs and deduced their length distribution. The results are shown in Figure 1B. We find that ideal ORFs in the yeast (Figure 1A) and ideal ORFs in stochastic sequences (Figure 1B) show the same length distribution in the range <200 amino acids, and both can be written as a

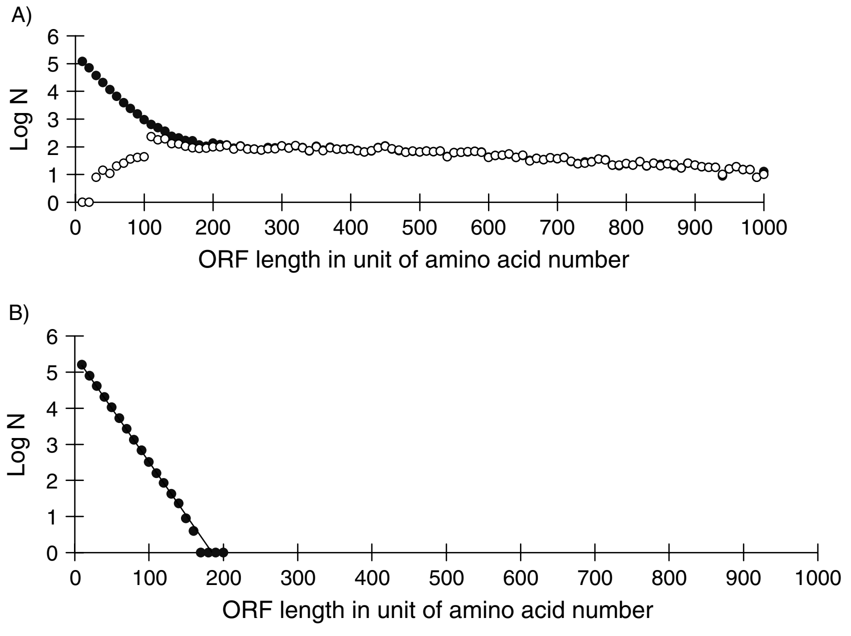

**Figure 1.** (A) The number of ORFs (logarithmic scale, i.e. log *N*), in each length interval of 10 amino acids, against ORF length, *l* (in units of amino acid number) for the yeast genome. The open circles (○) indicate the number of intron-less ORFs in the database and the filled circles (●) indicate the number of ideal ORFs (see Methods). The differences between two numbers are very small, and they overlap each other as ORF length becomes greater than 200. To improve clarity, the filled circles are omitted in this range, and only open circles are shown. (B) The number of ideal ORFs (logarithmic scale, i.e. log *N*), in each length interval of 10 amino acids, against ORF length, *l* (in units of amino acid number) for sequences stochastically generated under component constraint (see Methods)

Copyright © 2003 John Wiley & Sons, Ltd. 

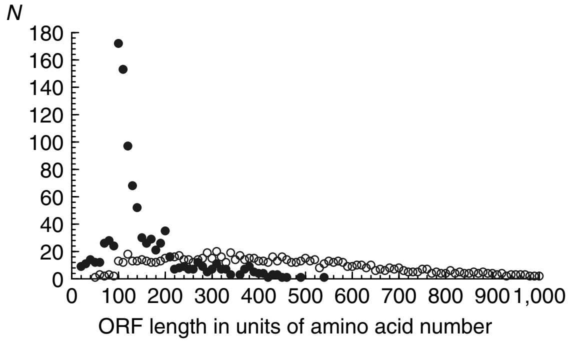

**Figure 2.** The size distributions of ORFs with IHI > 14, and of ORFs with IHI ≤ 14, in the yeast genome. In each case, the number of ORFs (*N*), in each length interval of 10 amino acids, are plotted against ORF length (in units of amino acid number). The total number of ORFs in all lengths has been normalized to 1000. The open circles (○) refer to the relative number of ORFs with IHI > 14, the filled circles (●) represent the relative number of ORFs with IHI ≤ 14

linear regression equation:

$$\log_{10} N = al + b \qquad (7)$$

where $N$ = the number of ORFs in a length interval of 10 amino acids and $l$ = ORF length in units of amino acid number. For yeast $a = -0.0231$, $b = 5.26$ and the correlation $R^2 = 0.996$ (99% confidence level); for stochastic sequence $a = -0.0297$, $b = 5.48$ and correlation $R^2 = 0.992$ (99% confidence level). The coincidence between these two distributions of ORF length agrees with the previously proposed random occurrence of short ORFs in this size range in *S. cerevisiae* (Dujon, 1996).

Further, we calculated the size distribution of all intron-less ORFs in the databases with IHI > 14 and IHI ≤ 14, respectively. The results are shown in Figure 2. As compared with IHI > 14 ORFs, the size distribution of IHI ≤ 14 ORFs is marked by a sharp maximum at short lengths, which seems related to the high distribution of ideal ORFs in this range for random sequence. It supports the assumption that many IHI ≤ 14 ORFs may be spurious.

### Online supplement

Supplementary material for this study can be found at **http://www.interscience.wiley.com/jpages/1531-6912/sites.html**.

### Acknowledgements

This work was supported by the National Science Foundation of China. The authors are grateful to the referees for language corrections and important suggestions on the manuscript.

### References

Blandin G, *et al.* 2000. Genomic exploration of the Hemiascomycetous yeasts: 4. The genome of *Saccharomyces cerevisiae* revised. *FEBS Lett* **487**: 31–36.

Burge CB, Karlin S. 1997. Prediction of complete gene structures in human genomic DNA. *J Mol Biol* **268**: 78–94.

Burge CB, Karlin S. 1998. Finding the genes in genomic DNA. *Curr Opin Struct Biol* **8**: 346–354.

Burset M, Guigo R. 1996. Evaluation of gene structure prediction programs. *Genomics* **34**: 353–367.

Claverie JM. 1997. Computational methods for the identification of genes in vertebrate genomic sequences. *Hum Mol Genet* **6**: 1735–1744.

Cliften PF, *et al.* 2001. Surveying *Saccharomyces* genomes to identify functional elements by comparative DNA sequence analysis. *Genome Res* **11**: 1175–1186.

Dujon B. 1996. The yeast genome project: what did we learn. *Trends Genet* **12**: 263–270.

Dujon B, Alexandraki D, Andre B, *et al.* 1994. Complete DNA sequence of yeast chromosome X.I. *Nature* **369**: 371–378.

Feller W. 1971. *An Introduction to Probability Theory and Its Applications*, 2nd edn, vol 2. Wiley: New York.

Fickett JW. 1982. Recognition of protein coding regions in DNA sequences. *Nucleic Acids Res* **10**: 5303–5318.

Fickett JW, Tung CS. 1992. Assessment of protein coding measures. *Nucleic Acids Res* **20**: 6441–6450.

Fukuda Y, Washio T, Tomita M. 1999. Comparative study of overlapping genes in the genomes of *M. genitalium* and *M. pneumoniae*. *Nucleic Acids Res* **27**: 1847–1853.

Gelfand MS, Mironov AA, Pevzner PA. 1996. Gene recognition via spliced sequence alignment. *Proc Natl Acad Sci USA* **93**: 9061–9066.

Goffeau A, Barrel BG, Bussey H, *et al.* 1996. Life with 6000 genes. *Science* **274**: 546.

Guigo R, Agarwal P, Abril JF, *et al.* 2000. An assessment of gene prediction accuracy in large DNA sequences. *Genom Res* **10**: 1631–1642.

International Human Genome Sequencing Consortium. 2001. Initial sequencing and analysis of the human genome. *Nature* **409**: 860–921.

Krogh A, Mian IS, Haussler D. 1994. A hidden Markov model that finds genes in *E. coli* DNA. *Nucleic Acids Res* **22**: 4768–4778.

Lee WJ, Luo LF. 1997a. Inhomogeneity analysis on DNA sequence. In *Collected Works on Theoretical Biophysics*, Luo LF (ed.). Inner Mongolia University Press: Hohhot; 321–324.

Lee WJ, Luo LF. 1997b. The periodicity of base correlation in nucleotide sequence. *Phys Rev E* **56**: 848–851.

Lobzin VV, Chechetkin VR. 2000. Order and correlations in genomic DNA sequences. The spectral approach. *Physics Uspekhi* **43**: 55–78.

Copyright © 2003 John Wiley & Sons, Ltd.

Luo LF, Li H. 1991. The statistical correlation of nucleotides in protein-coding DNA sequences. *Bull Math Biol* **53**: 345–353.

Mackiewicz P, Kowalczuk M, Gierlik A, *et al.* 1999. Origin and properties of non-coding ORFs in the yeast genome. *Nucleic Acids Res* **27**: 3503–3509.

Mewes HW, Albermann K, Bahr M, *et al.* 1997. Overview of the yeast genome. *Nature* **387**(suppl): 7–8.

Staden R. 1984. Measurement of the effects that coding for a protein has on a DNA sequence and their use for finding genes. *Nucleic Acids Res* **12**: 551–567.

Stormo GD. 2000. Gene-finding approaches for eukaryotes. *Genom Res* **10**: 394–397.

Termier M, Kalogeropoulos A. 1996. Discrimination between fortuitous and biologically constrained ORFs in DNA sequences of *Saccharomyces cerevisiae*. *Yeast* **12**: 369–384.

Velculescu VE, Zhang L, Zhou W, *et al.* 1997. Characterization of the yeast transcriptome. *Cell* **88**: 243–251.

Wood V, Rutherford AI, Rajandream MA, *et al.* 2001. A re-annotation of the *Saccharomyces cerevisiae* genome. *Comp Funct Genom* **2**: 143–154.

Zhang CT, Wang J. 2000. Recognition of protein coding genes in the yeast genome at better than 95% accuracy based on Z curve. *Nucleic Acids Res* **28**: 2804–2814.

Copyright © 2003 John Wiley & Sons, Ltd. 
